# Sensitive Fluorescent Sensor for Recognition of HIV-1 dsDNA by Using Glucose Oxidase and Triplex DNA

**DOI:** 10.1155/2018/8298365

**Published:** 2018-04-01

**Authors:** Yubin Li, Sheng Liu, Liansheng Ling

**Affiliations:** ^1^College of Chemistry and Environment, Guangdong Ocean University, Zhanjiang 524088, China; ^2^School of Chemistry, Sun Yat-Sen University, Guangzhou 510275, China

## Abstract

A sensitive fluorescent sensor for sequence-specific recognition of double-stranded DNA (dsDNA) was developed on the surface of silver-coated glass slide (SCGS). Oligonucleotide-1 (Oligo-1) was designed to assemble on the surface of SCGS and act as capture DNA, and oligonucleotide-2 (Oligo-2) was designed as signal DNA. Upon addition of target HIV-1 dsDNA (Oligo-3•Oligo-4), signal DNA could bind on the surface of silver-coated glass because of the formation of C•GoC in parallel triplex DNA structure. Biotin-labeled glucose oxidase (biotin-GOx) could bind to signal DNA through the specific interaction of biotin-streptavidin, thereby GOx was attached to the surface of SCGS, which was dependent on the concentration of target HIV-1 dsDNA. GOx could catalyze the oxidation of glucose and yield H_2_O_2_, and the HPPA can be oxidized into a fluorescent product in the presence of HRP. Therefore, the concentration of target HIV-1 dsDNA could be estimated with fluorescence intensity. Under the optimum conditions, the fluorescence intensity was proportional to the concentration of target HIV-1 dsDNA over the range of 10 pM to 1000 pM, the detection limit was 3 pM. Moreover, the sensor had good sequence selectivity and practicability and might be applied for the diagnosis of HIV disease in the future.

## 1. Introduction

Double-stranded structure, reported in 1953 [1], is the natural state of DNA. Meanwhile, double-stranded DNA (dsDNA) detection is of particular importance in gene therapy, diagnosis, and monitoring fatal infections caused by viruses and diseases that are associated with genetic alterations [2–8]. Routine protocols for sequence-specific recognition of dsDNA, for instance, are performed by using zinc finger DNA-binding proteins [9, 10], polyamides [11, 12], and triplex-forming oligonucleotides [13–17]. Triplex-forming oligonucleotide-based methods commonly require the protonation of cytosine for the formation of C•GoC (“•” denotes the Watson–Crick bond, and “o” denotes the Hoogsteen bond) in parallel triplex DNA structure. This has the performance of binding the major groove of dsDNA and exerting high sequence specificity, which have been extensively used for the analysis of dsDNA [18].

Enzymes are highly applicable in biosensors as recognition and signaling elements for the detection of specific molecules due to the features such as high sensitivity and good selectivity [19]. Given these favorable characteristics, glucose oxidase (GOx) not only can catalyze the oxidation of glucose but also is one of the cheapest and most stable redox enzymes. Additionally, on one hand, GOx has been used in constructing electrochemical [20–23], fluorescence [24–26],and colorimetric [27, 28] sensors for glucose. On the other hand, GOx is conjugated for recognition of biomolecules and acted as an amplifying label, which is successfully applied to establish sensors for proteins [29, 30] and DNA [31].

Herein, we explore the possibility to develop a fluorescent sensor for sensitive and sequence-specific detection of target dsDNA. The sequence of target dsDNA is from site 7960 to site 7991 of longest homopurine-homopyrimidine duplex strand in the human immunodeficiency virus 1 (HIV-1) dsDNA gene [32, 33]. The enzyme immunoassay (EIA) test has been authorized by Food and Drug Administration to recognize HIV by measuring humans' antibody response. However, there is a 25-day infectious window period for HIV EIAs [34]. The proposed method is expected to shorten this period. Besides, there are a lot of sensitive methods to detect HIV-1 RNA [35, 36], yet HIV-1 RNA may be cleaved during the process of sample preparation, which limits their application in real samples. HIV-1 RNA is integrated into the host gene forming double-stranded DNA in 3 days. Thus, the identification of HIV-1 dsDNA may play a significant role here. PCR-based tests for the detection of HIV-1 DNA are sensitive and specific, but their application in resource-limited areas is hindered due to the time consumingness of nucleic acid purification and the requirement of skilled processing and costly reagents and equipment [37]. Therefore, it is urgent to develop a new method to replace the PCR-based tests. This protocol takes advantage of the amplification property of GOx, the capture DNA is assembled on the surface of SCGS, and signal DNA is designed to conjugate with GOx. Upon addition of target HIV-1 dsDNA, GOx could be immobilized on the surface of SCGS. Thereby, the concentration of target HIV-1 dsDNA controlled the number of bound GOx, which could be detected with the fluorescence of oxidized HPPA. These methods have been applied to develop sensors for sequence-specific recognition of dsDNA based upon triplex formation [13–17]. Compared with the above methods, the proposed method is simpler, convenient, and time-saving.

## 2. Experimental

### 2.1. Materials, Chemicals, and Instrumentation

Tri-(2-carboxyethyl) phosphine (TCEP) and bovine serum albumin (BSA) were purchased from Sigma-Aldrich (USA). Amicon filtration device and EZ-link sulfo-NHS-biotinylation kit were purchased by Thermo Fisher Scientific Inc. (USA). Horseradish peroxidase (HRP), streptavidin (SA), glucose oxidase (GOx), 3-(*p*-hydroxyphenyl)-propanoic acid (HPPA), DNase I, ammonia (25%), silver nitrate, and glucose were purchased from Sinopharm Chemical Reagent Co., Ltd. (Beijing, China). All oligonucleotides (Table 1) were synthesized and purified by Sangon Bioengineering Technology and Services Co., Ltd. (Shanghai, China). They were dissolved in PBS buffer. All chemicals were of analytical reagent grade. All solutions were prepared with ultrapure water.

PBS buffer (pH 6.0; 100 mM Na_2_HPO_4_, 100 mM NaH_2_PO_4_, and 100 mM NaNO_3_) and Tris-HAc buffer (10 mM, pH 7.5; 2.5 mM Mg(NO_3_)_2_ and 0.5 mM Ca(NO_3_)_2_) were prepared for research.

The pH values of the solutions were measured by using a pHS-3E digital pH meter (Shanghai Leici Instrument Plant, China). The implementation of fluorescence measurements was with the help of RF-5301PC spectrofluorometer (Shimadzu, Japan), and the slit width was 5.0 nm. In addition, the excitation spectrum was set at 320 nm, while the emission spectra were collected from 380 nm to 450 nm. The circular dichroism spectra (CD spectra) were measured by using J-810 circular dichroism spectrum (Shimadzu, Japan), the range of emission wavelength were from 200 nm to 300 nm, the scanning speed was 100 nm·min^−1^, the response time was 1 second, and its bandwidth was 1.71 nm. In addition, for the continuous scanning mode, the spectral scanning number was 3.

### 2.2. Preparation of SCGS

According to Li et al. [38], the SCGSs were prepared by the traditional silver mirror reaction.

### 2.3. Preparation of Biotin-Modified Glucose Oxidase

Biotin can be connected to glucose oxidase (GOx) by the cross-linking agent sulfo-NHS-biotin, and the biotin-labeled glucose oxidase (biotin-GOx) was prepared as in [38].

### 2.4. Immobilization of Oligo-1 on the SCGS

According to the reported methods, sulfhydryl capture DNA (SH-Oligo-1) can be immobilized on the SCGS [39, 40]. The SCGS was placed in PBS buffer which contains 1.4 *μ*M Oligo-1. In order to eliminate free Oligo-1, the modified silver-coated glass was washed twice by using the same buffer.

### 2.5. Fabrication of the Sensor

Firstly, the capture DNA-modified SCGS was submerged into different concentrations of target HIV-1 dsDNA (Oligo-3•Oligo-4) solution for 1 hour. Then, the slide was immersed into 50 nM signal DNA solution for another 1 hour. After washing twice by using PBS buffer, the slide was immersed in 3% BSA solution for 20 minutes to block possible remaining active sites. Then, the slide was dripped into 800 ng·mL^−1^ streptavidin solution and 50 *μ*g·mL^−1^ biotin-GOx solution, respectively, for 10 minutes. The procedures mentioned above were carried out in PBS buffer at room temperature. After washing twice by using PBS buffer, the slide was immersed into Tris-HAc buffer containing 50 U DNase I for 1 hour at 37°C, and the slide was taken out. Then, 50 mM glucose was injected into the mixture for 2 hours at 37°C to yield H_2_O_2_. After that, 200 *μ*M HPPA and 20 ng·mL^−1^ HRP were added into the abovementioned solution at the same time in darkness for 30 minutes at 37°C. Eventually, fluorescent spectra of the oxidative products of HPPA were recorded by using a RF-5301PC spectrofluorometer [41].

### 2.6. Preparation of Human Sera Samples

Human sera was obtained from Guangdong Ocean University Campus Hospital (Zhanjiang, China), before the test with calf thymus DNA interference and recovery experiment in human sera, and each sample was dealt with the Amicon filtration device 10,000 to remove small molecules. And the calf thymus DNA was digested into smaller fragments before using.

## 3. Results and Discussion

### 3.1. Design of the Sensor

**Figure 1:**
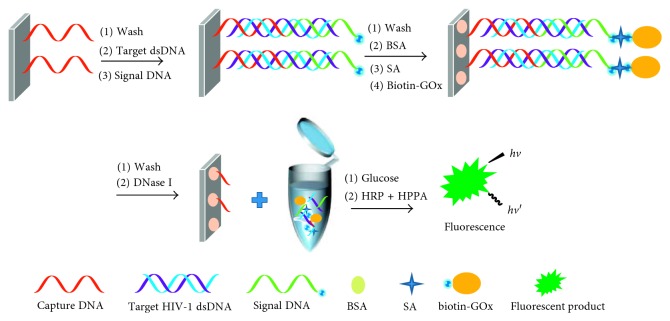
The scheme for fluorescent sensor for sequence-specific recognition of target dsDNA by using capture DNA and amplification by using glucose oxidase.

The scheme of the sensor is depicted in Figure 1. Capture DNA is for the purpose of aggregating on the surface of SCGS by use of Ag–S bond. Signal DNA can bind on the surface of SCGS due to the addition of target HIV-1 dsDNA. GOx can also bind to signal DNA by streptavidin-biotin bond after addition of streptavidin and biotin-GOx. And the concentration of GOx immobilizing on the surface of SCGS is dependent on that of target HIV-1 dsDNA. In order to avoid the nonspecific adsorption of streptavidin and GOx, DNase I is used to cleave the DNA strand from the surface of SCGS, and the bound glucose oxidase is transferred into the buffer. Thus, the concentration of target dsDNA is transduced into the concentration of H_2_O_2_ which is the oxidative product of glucose in the presence of GOx. Then, HPPA can be oxidized into the fluorescent product by H_2_O_2_ under the catalysis of HRP. Finally, the concentration of target HIV-1 dsDNA was estimated with the fluorescence intensity of oxidized HPPA.

### 3.2. Fluorescence Spectrum

**Figure 2:**
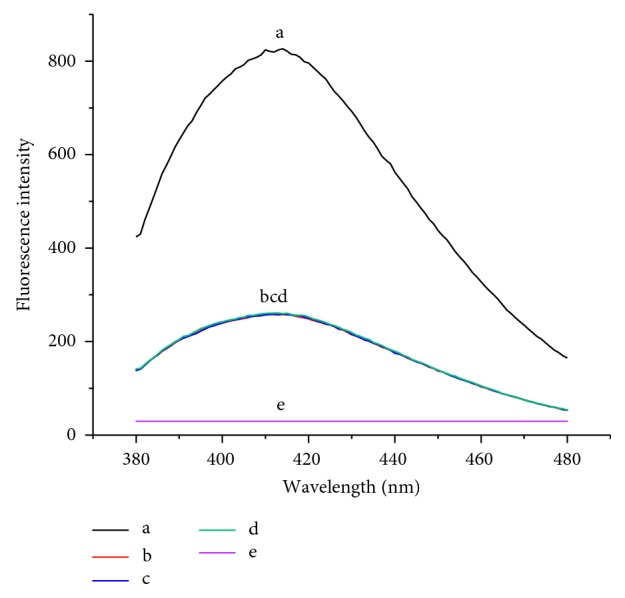
Fluorescence spectra of different mixtures under the same process. (a) Capture DNA + signal DNA + biotin-GOx + target HIV-1 dsDNA; (b) capture DNA + signal DNA + biotin-GOx; (c) target HIV-1 dsDNA + signal DNA + biotin-GOx; (d) capture DNA + target HIV-1 dsDNA + biotin-GOx; (e) capture DNA + target HIV-1 dsDNA + signal DNA.

To examine the feasibility of the sensor, the fluorescence signal is illustrated in Figure 2. It was demonstrated that there was no fluorescence without biotin-GOx (curve e). Moreover, the fluorescence intensities of the mixture were weak in the absence of target HIV-1 dsDNA (curve b), capture DNA (curve c), or signal DNA (curve d). However, the intensity increases were enhanced dramatically with the addition of 1000 pM target HIV-1 dsDNA (curve a).

### 3.3. Circular Dichroism (CD) Spectroscopy of the Sensor

**Figure 3:**
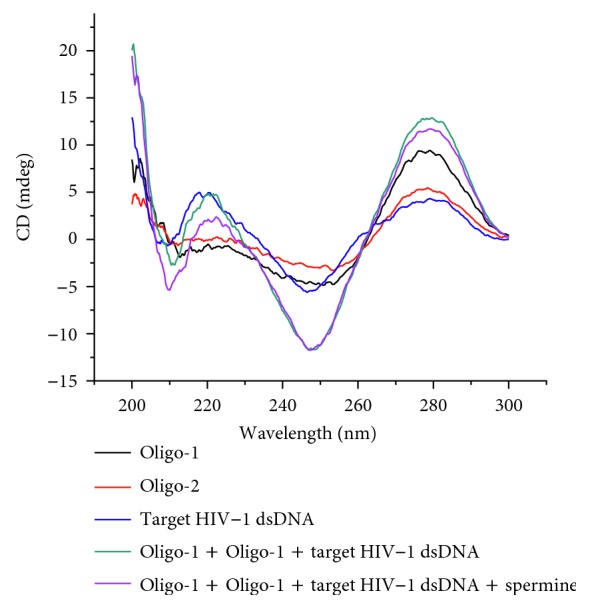
Circular dichroism (CD) spectroscopy of the sensor.

Circular dichroism (CD) spectroscopy is an effective tool and has been extensively used in the study of DNA structure.  Figure 3 demonstrates that the CD spectroscopy of capture DNA (Oligo-1) was almost the same as that of signal DNA (Oligo-2). This was the classic spectroscopy of single-stranded DNA which had a weak positive Cotton effect peak around 275 nm and a weak negative Cotton effect peak at 249 nm. There was a strong peak at 218 nm, which indicated the helicity of dsDNA [42]. When target HIV-1 dsDNA was added into Oligo-1 and Oligo-2, the negative peak of 210 nm increased apparently, which was the marker of the triplex DNA [43]. Upon addition of spermine, the negative peak of 210 nm increased obviously. This phenomenon explained that spermine can increase the stability of triplex DNA. Overall, these results were consistent with that of the absorption spectra.

### 3.4. Optimization of the Experimental Conditions

**Figure 4:**
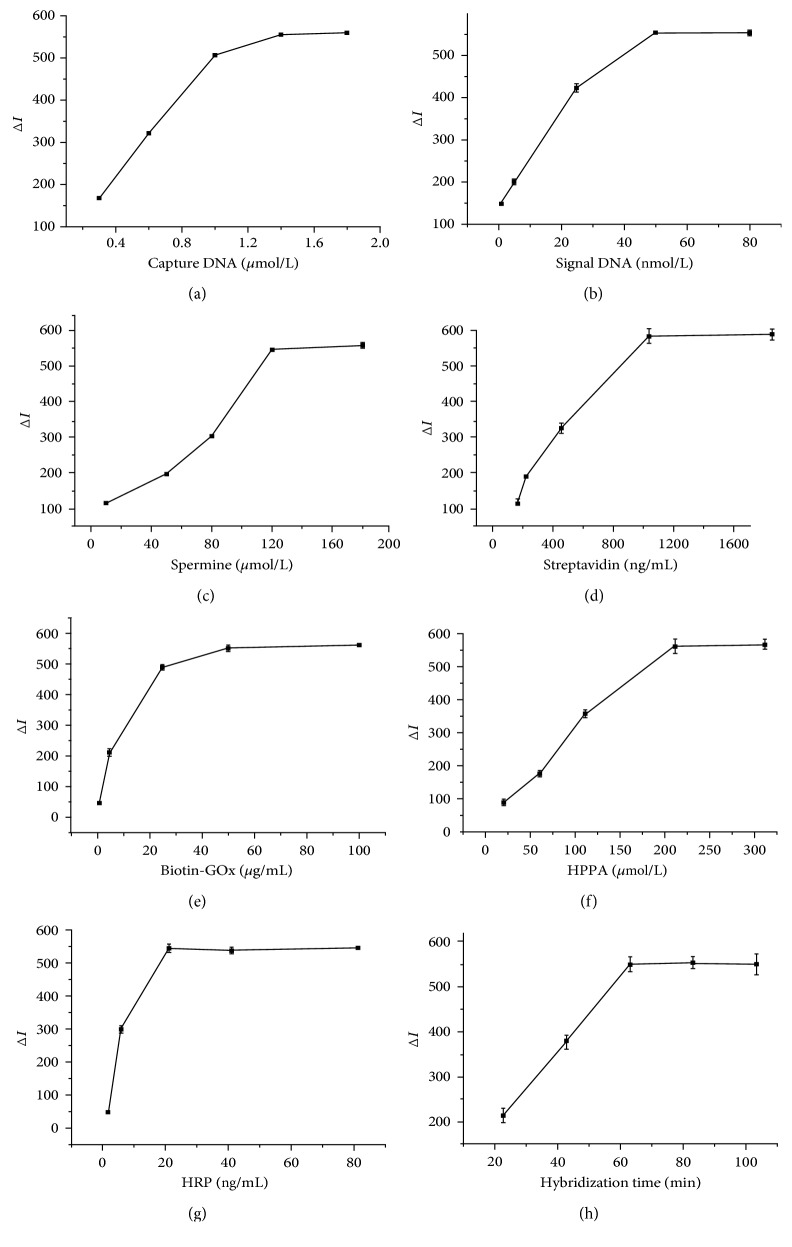
(a) Effects of capture DNA concentrations; (b) effects of signal DNA concentrations; (c) effects of spermine concentrations; (d) effects of streptavidin concentrations; (e) effects of biotin-GOx concentrations; (f) effects of HPPA concentrations; (g) effects of HRP concentrations; (h) effects of time of hybridization. When each of these factors is optimized, the other factors maintain optimal conditions (1.4 *μ*M capture DNA, 50 nM signal DNA, 120 *μ*M spermine, 800 ng·mL^−1^ SA, 50 *μ*g·mL^−1^ biotin-GOx, 200 *μ*M HPPA, 20 ng·mL^−1^ HRP, and 60 min of time of hybridization).

To gain the optimal results, the following factors that affected the method performance were optimized, including the concentration of capture DNA, signal DNA, spermine, streptavidin, biotin-GOx, HPPA, HRP, and hybridization time. The optimal conditions were selected by obtaining the maximum change of fluorescence intensity (Δ*I*). Δ*I* was defined as *I*_target_ − *I*_blank_, where *I*_target_ represents the fluorescence intensity of the mixture that contains target HIV-1 dsDNA and *I*_blank_ denotes the fluorescence intensity in the absence of target HIV-1 dsDNA. A good detection performance was obtained when 1.4 *μ*M capture DNA, 50 nM signal DNA, 120 *μ*M spermine, 800 ng·mL^−1^ SA, 50 *μ*g·mL^−1^ biotin-GOx, 200 *μ*M HPPA, and 20 ng·mL^−1^ HRP and 60 min of time of hybridization were used (Figure 4). The temperature can affect the formation of dsDNA; the effect of temperature is shown in Figure \ S1. When each of these factors is optimized, the other factors maintain optimal conditions.

### 3.5. Calibration Curve and Absorption Spectra for Target dsDNA

**Figure 5:**
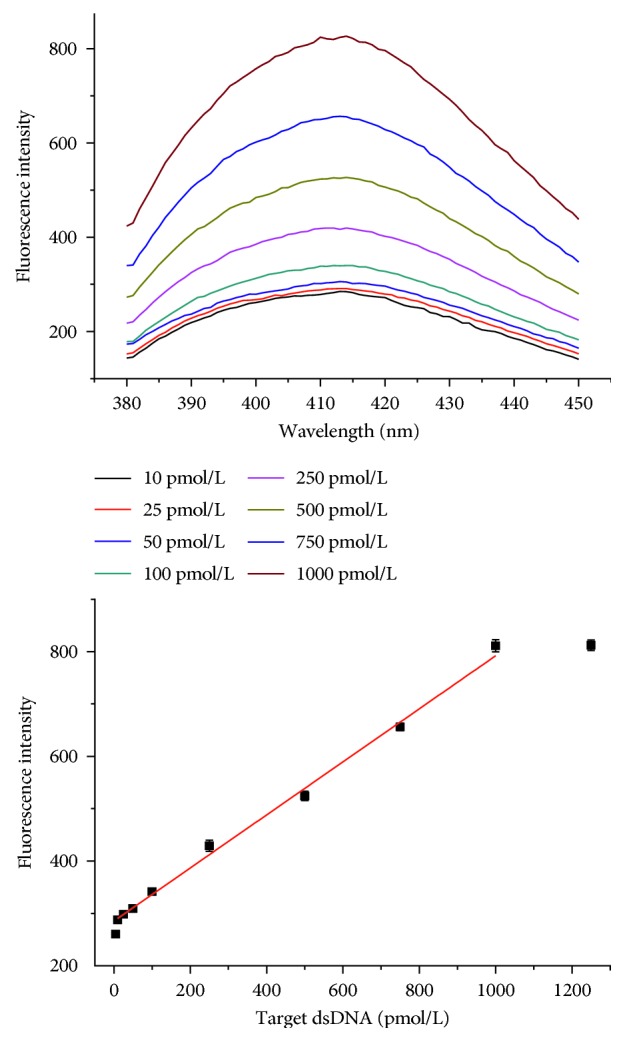
Calibration curve and absorption spectra for various concentrations of target dsDNA.

The relationship between the fluorescence intensity and the concentration of target HIV-1 dsDNA was studied under the optimum conditions. As shown in Figure 5, the fluorescence intensity increased with the concentration of target dsDNA over the range of 10 pM to 1000 pM, with a linear regression equation of *I* = 0.507 *C* + 265.54 (*C*: pM, *r* = 0.996, where *C* denotes the concentration of target HIV-1 dsDNA and *I* represents the fluorescence intensity) and a detection limit of 3 pM, which was obtained from the equation of DL = 3б/slope. The comparison with other methods is listed in Table 2.

### 3.6. Sequence Selectivity of the Sensor

**Figure 6:**
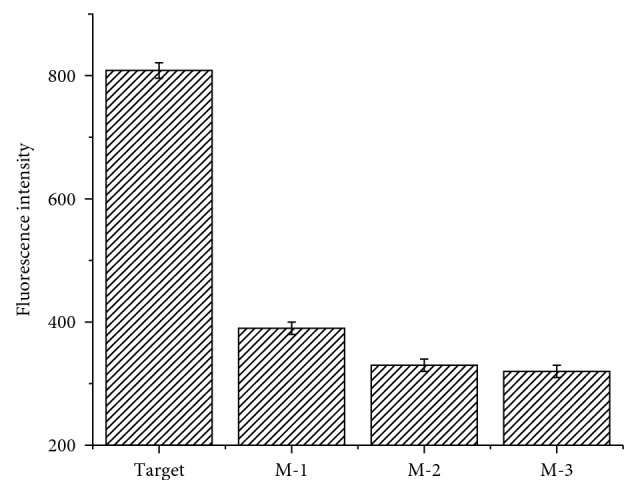
Sequence selectivity of the sensor. The concentration of target dsDNA, M-1, M-2, and M-3 was 1000 pM. Every point is the mean of three measurements. The error bar was the standard deviation.

For the purpose of exploring the sequence selectivity of the sensor, the sequence selectivity was investigated by means of replacing the target HIV-1 dsDNA with other three complementary dsDNAs, respectively. As shown in Figure 6, the intensity for target HIV-1 dsDNA was about 810, while that for single-base mismatched strand M-1 (Oligo-5•Oligo-6) and the two-base mismatched strands M-2 and M-3 (Oligo-7•Oligo-8 and Oligo-9•Oligo-10) were about 400, 320, and 315, respectively. These results illustrated that the proposed sensor had good sequence selectivity for target HIV-1 dsDNA.

### 3.7. Detection of Target HIV-1 dsDNA in Human Serum

**Figure 7:**
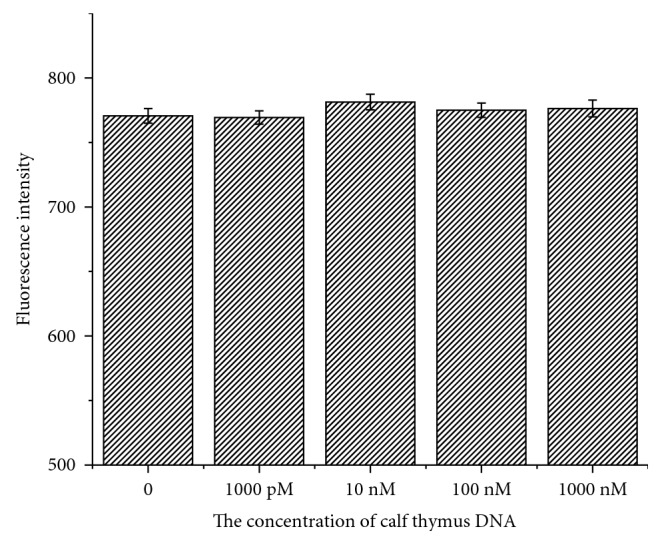
Effect of calf thymus DNA on the fluorescence of 1.0 × 10^−9^ M HIV-1 dsDNA.

To investigate the effect of other genes on the proposed strategy, test with calf thymus DNA interference aimed to recognize target dsDNA in the large amount of other dsDNA. As shown in Figure 7, there was little significant change in the fluorescence intensity with the addition of the concentration of calf thymus DNA increasing from 1000 pM to 1000 nM by using the proposed strategy. These results indicated that the proposed strategy could overcome the effect of other similar homopurine homopyrimidine sequences in the genome completely, and the effect of other similar homopurine homopyrimidine sequences in the genome could be ignored in the proposed method.

To assess the analytical application of the sensor in clinical specimens, the method was used to detect target HIV-1 dsDNA in the human serum. Since no HIV-1 dsDNA was found from the healthy volunteers' human serum, addition and recovery experiment was executed to evaluate the application of the sensor, and 1000 nM digested calf thymus DNA fragments were added into the serum as control DNA. As demonstrated in Table 3, 5.0 × 10^−11^−5.0 × 10^−9^ M of target HIV-1 dsDNA was added into each human serum, the recovery ranged from 93.0% to 103.2%, and the relative standard deviation values were in the range of 4.2%–7.3%, which meant that the application of the proposed sensor in real sample was possible.

## 4. Conclusions

In conclusion, through the amplifying property of GOx, a sensitive fluorescent sensor for sequence-specific recognition of target dsDNA was established. The target dsDNA which was selected from site 7960 to site 7991 of the HIV-1 dsDNA gene was designed as target HIV-1 dsDNA. Capture DNA was immobilized on the surface of SCGS by Ag–S bond, and with the help of biotin-SA, signal DNA can be conjugated with SA-biotin-GOx. Upon addition of target HIV-1 dsDNA, the GOx could be immobilized on the surface of silver-coated glass because of the formation of C•GoC in parallel triplex DNA structure. Thus, the concentration of target dsDNA controlled the number of bound GOx, which could be detected with the fluorescence of oxidized HPPA. Under the optimum conditions, the fluorescence intensity was proportional to the concentration of target HIV-1 dsDNA over the range from 10 pM to 1000 pM, with a detection limit of 3 pM. In addition, the sensor is target specific and is practicable. This is the first report of GOx amplification for sequence-specific recognition of dsDNA, and this assay might open a new avenue for applying in the diagnosis of HIV disease in the future.

## Figures and Tables

**Table 1:** Sequences of oligonucleotides.

Probes	Name	Sequence
Oligo-1	Capture DNA	5′-SH-(T)_12_ CTT CCT TAT CTT CTT C-3′
Oligo-2	Signal DNA	5′-TTC CAC CTC TCT CTC T (T)_12_-biotin-3′
Oligo-3	Target dsDNA	5′-TCT CTC TCT CCA CCT TCT TCT TCT ATT CCT TC-3′
Oligo-4		5′-GAA GGA ATA GAA GAA GAA GGT GGA GAG AGA GA-3′
Oligo-5	M-1 dsDNA	5′-TCT CTC TCT ACA CCT TCT TCT TCT ATT CCT TC-3′
Oligo-6		5′-GAA GGA ATA GAA GAA GAA GGT GTA GAG AGA GA-3′
Oligo-7	M-2 dsDNA	5′-TCT CTC TCT ACA CCT TCT TCT TCC ATT CCT TC-3′
Oligo-8		5′-GAA GGA ATG GAA GAA GAA GGT GTA GAG AGA GA-3′
Oligo-9	M-3 dsDNA	5′-TCT CCC TCT ACA CCT TCT TCT TCT ATT CCT TC-3′
Oligo-10		5′-GAA GGA ATA GAA GAA GAA GGT GTA GAG GGA GA-3′

**Table 2:** Comparison of our method with other methods for the determination of dsDNA.

Method	Analytical range	LOD	Application to samples	Reference
Polyamide microarrays method	1 nM–6 *μ*M	1 nM	No	[12]
Strand-displacement amplification method	1 pM–250 pM	0.4 pM	No	[13]
Dynamic light-scattering method	59 pM–4061 pM	59 pM	No	[15]
Molecular beacon method	0.75 nM–50 nM	0.69 nM	No	[17]
Nicking enzyme amplification method	100 pM–200 nM	66 pM	Yes	[33]
Glucose oxidase amplification method	10 pM–1000 pM	3 pM	Yes	This work

**Table 3:** Recoveries of HIV-1 dsDNA from the spiked human serum samples with 1000 nM digested calf thymus DNA fragments.

Serum samples	Added HIV dsDNA (M)	Founded HIV dsDNA (M)	Recovery (%)	Relative standard deviation (%)
1	5.0 × 10^−11^	5.16 × 10^−11^	103.2	7.3
2	1.0 × 10^−10^	1.13 × 10^−10^	113.0	6.8
3	5.0 × 10^−10^	4.65 × 10^−10^	93.0	6.6
4	1.0 × 10^−9^	0.98 × 10^−8^	98.0	4.2

The values shown here are the average values from three measurements.

## Supplementary Materials

Supplementary Materials Figures S1: Effects of temperature.
