# Supplementary material for: Sensitive Fluorescent Sensor for Recognition of HIV-1 dsDNA by Using Glucose Oxidase and Triplex DNA
Source: J Anal Methods Chem. 2018 Apr 1;2018:8298365. doi: 10.1155/2018/8298365 (PMC5901486; doi:10.1155/2018/8298365)
Supplement: Supplementary Materials — Figures S1: Effects of temperature. [file 8298365.f1.docx]

**Sensitive fluorescent sensor for recognition of HIV-1 dsDNA by using Glucose Oxidase and triplex DNA**

Yubin Li*^,1^, Sheng Liu^1^, Liansheng Ling**^,2^

1. College of Chemistry and Environment, Guangdong Ocean University, Zhanjiang, 524088, P. R. China.

2. School of Chemistry, Sun Yat-Sen University, Guangzhou 510275, P. R. China


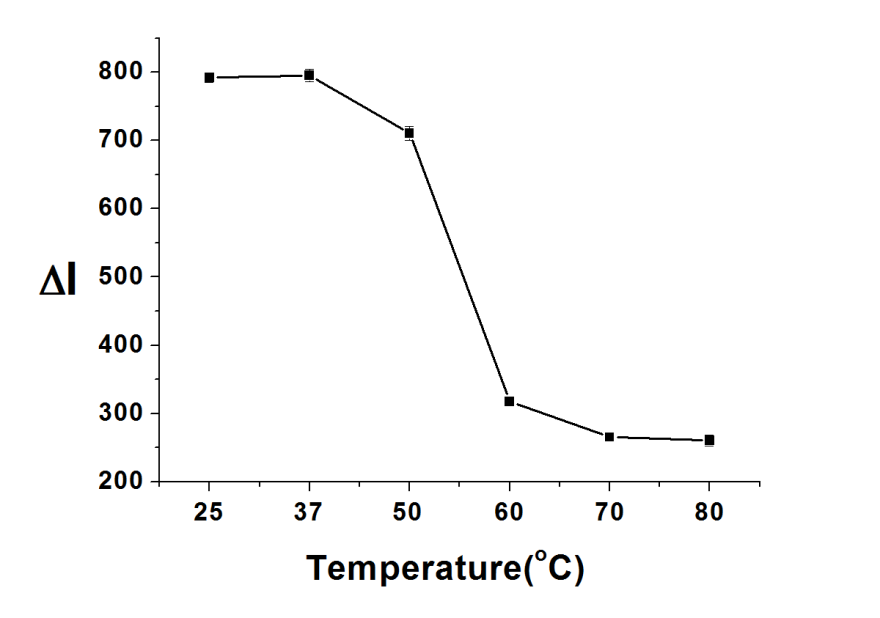


Figure S1. Effects of temperature
